# Dispersion-Stable Carboxymethyl Cellulose/Single-Walled Carbon Nanotube Composite for Water-Processed Organic Thermoelectrics

**DOI:** 10.3390/ma18020337

**Published:** 2025-01-13

**Authors:** Jaehee Jang, Hyejeong Yeom, Sujong Chae, Seyoung Kee

**Affiliations:** 1 Department of Polymer Engineering, Pukyong National University, Busan 48513, Republic of Korea; 2 Division of Applied Chemical Engineering, Pukyong National University, Busan 48513, Republic of Korea

**Keywords:** organic thermoelectrics, organic thermoelectric generator, single-walled carbon nanotube, carboxymethyl cellulose, dispersion-stable

## Abstract

Carbon nanotubes (CNTs) have drawn great attention as promising candidates for realizing next-generation printed thermoelectrics (TEs). However, the dispersion instability and resulting poor printability of CNTs have been major issues for their practical processing and device applications. In this work, we investigated the TE characteristics of water-processable carboxymethyl cellulose (CMC) and single-walled CNT (SWCNT) composite. The microscopic analyses indicated that the CMC-incorporated SWCNT dispersions produced uniform and smooth TE films, capable of ensuring reliable TE performance. The resulting composite films provided a low temperature power factor of 73 μW m^−1^ K^−2^ with a high electrical conductivity of ≈1600 S cm^−1^ and a Seebeck coefficient of ≈21 µV K^−1^. Moreover, the composite films possessed low thermal conductivity of ≈25 W m^−1^ K^−1^, significantly lower than that of pure SWCNTs, with a maximum figure of merit of 1.54 × 10^−3^ at 353.15 K. Finally, we successfully demonstrated water-processed organic TEGs using CMC/SWCNT films as a p-type component. This work could offer valuable insights to support the development of printable organic-based TE materials and devices.

## 1. Introduction

Thermoelectrics (TEs) based on π-conjugated organic materials possessing excellent mechanical deformability and electronic/electrical tunability have attracted great attention for use in applications such as wearable power generation, cooling systems in medical devices, and TE sensor devices [1,2,3,4,5]. The unique solution processibility of organic TE materials enables large-area printing as well as highly integrated micro fabrication of TE generators (TEGs), namely, so-called ‘printed TEs’ [1,6,7]. Furthermore, their adjustable solubility via structural modification and chemical functionalization enables successful eco-friendly processing in halogen-free aqueous media [8]. Despite the given advantages, current TEGs have been developed using mainly bismuth telluride (BiTe) and its alloys, which exhibit outstanding TE performance with a *ZT* exceeding 1.0 at around 300 K [9,10]. The performance of TE materials is evaluated according to the following dimensionless figure of merit (*ZT*):

$$ZT=\frac{S^{2}\sigma }{\kappa }T$$

where *S*, *σ*, *T*, and *κ* are Seebeck coefficient, electrical conductivity, absolute temperature, and thermal conductivity, respectively. However, their reliance on high-temperature sintering processes and their rigid, heavy nature limits their applicability in printed and flexible lightweight TEGs. To realize such next-generation TE technologies, organic printable TE materials with high *ZT* values comparable with those of BiTe-based TE materials (i.e., *ZT* ≈ 1.0 at room temperature) have high research potential.

Carbon nanotubes (CNTs) are a novel class of organic-based conductive nanomaterial, and their application in TEs has been a key research area for establishing high-performance organic TEs [11,12,13,14,15,16]. Interestingly, CNTs can act as both p-type and n-type elements, based on their wide electronic tunability depending on the type of dopants [17,18], which is an important beneficial factor for the fabrication of TEGs composed of solution-processed p–n pairs. However, unstable dispersion of CNTs arising from strong π–π aggregation in the solution state poses a significant challenge to their reliable processing and practical application. While dispersant-free techniques (e.g., ball milling and thermal dispersion) have been explored to address this issue, they often fail to achieve uniform distribution, highlighting the critical need for effective dispersion methods [19,20]. Such methods are essential for stabilizing CNTs in solution and ensuring consistent performance across various applications. Mechanical methods, including ultrasonic dispersion techniques, are widely employed to create high-energy environments that effectively disaggregate CNT agglomerates, resulting in a more uniform dispersion [21,22]. Covalent functionalization, involving the attachment of functional groups such as −COOH, −OH, and −NH_2_ to the CNT surface, significantly enhances their solubility in polar solvents and improves compatibility with various matrices [23,24]. Additionally, non-covalent functionalization using dispersants and surfactants, including poly(ethylene oxide) and polyvinyl alcohol, introduces steric or electrostatic repulsion, preserving the intrinsic properties of CNTs while effectively mitigating aggregation and stabilizing their dispersion in solution [23,25,26]. Composite formation strategies offer a versatile and efficient approach to achieving stable CNT dispersions by overcoming strong π–π interactions and van der Waals forces [27,28,29,30,31,32]. Furthermore, hydrogen bonding between functionalized CNTs and matrix polymers not only stabilizes the dispersion but also reinforces the mechanical and functional properties of the resulting composites, making this method highly advantageous for diverse applications [23,27,33,34].

In this research, we investigated the TE properties of dispersion-stable and water-processable carboxymethyl cellulose (CMC) and single-walled CNT (SWCNT) composite. The incorporation of the water-soluble CMC component as a matrix polyelectrolyte enabled stable dispersions of SWCNTs in water solvent. Atomic force microscope (AFM) and scanning electron microscope (SEM) analyses proved that the dispersed CMC/SWCNT composites were able to produce smooth thin and thick films. The resulting CMC/SWCNT films exhibited *σ* and *S* values of 1630 S cm^−1^ and 21 µV K^−1^, respectively, at low temperature, leading to a power factor (*PF*, *S*^2^*σ*) of 73 μW m^−1^ K^−2^. Moreover, a maximum *PF* of 113 μW m^−1^ K^−2^ was achieved at 353 K. We also found that the introduction of CMC significantly reduced the *κ* of pure SWCNTs, which resulted in a *κ* of 25 W m^−1^ K^−1^ at low temperature. As a result, using CMC/SWCNT films with promising TE properties, we demonstrated water-processed organic TEGs for printable TE applications.

## 2. Materials and Methods

Materials and samples: CMC/SWCNT (TUBALL BATT H_2_O with SWCNT 0.4 wt% and CMC 0.6 wt%) was supplied from OCSiAl Co. (Foetz, Grand-Duché de Luxembourg). For the preparation of both thin and thick films, CMC/SWCNT dispersions were diluted with deionized (DI) water (a weight ratio of 1 to 1) and the diluted solution was stirred at 500 rpm for over 3 h. For preparation of thin films, the diluted CMC/SWCNT dispersions were spin-coated on substrates and annealed at 130 °C for 10 min. The thick CMC/SWCNT films were prepared by drop-casting on substrates and then evaporating the water content at room temperature overnight, and finally annealing at 130 °C for 20 min. The glass substrates were pre-cleaned (in the order: detergent, DI water, acetone, isopropyl alcohol) and then treated with UV/O_3_ for 20 min. The pre-patterned chips for TE parameter measurements were treated only with UV/O_3_ for 10 min before solution deposition.

Device fabrication: For the fabrication of CMC/SWCNT-based TEGs, p-type CMC/SWCNT films were drop-cast over the entire glass substrates with a size of 5 × 5 cm^2^ (pre-cleaned and UV/O_3_-treated). The prepared CMC/SWCNT thick films (with the thickness in µm scale) were patterned by wiping them into the desired patterns and device configuration (each p-leg with a size of 2 × 10 mm^2^). Using silver paste, the eight p-legs were all electrically connected in series.

Characterization: The *σ*, *S*, and *κ* of CMC/SWCNT thick films (with the thickness in µm scale) were analyzed using Linseis TFA. All the measurements were implemented through the in-plane direction under a high vacuum (≈10^−6^ Torr). The thickness of films was characterized using a surface profiler (Alpha-Step IQ, KLA Tencor, Milpitas, CA, USA). AFM measurement was carried out using a Park Systems (Suwon, Republic of Korea) NX7 microscope in noncontact mode. SEM measurement was conducted using Mira 3 LMH, TESCAN (Brno, Kohoutovice Czech Republic), and a conductive Pt supporting layer (Merck Co., Darmstadt, Germany) was deposited on the samples before the measurements. Thermogravimetric analysis (TGA) was conducted using a TA Instruments SDT650 (New Castle, DE, USA) analyzer under a nitrogen atmosphere to evaluate the thermal stability of the composite films. The temperature was ramped from 25 °C to 1000 °C at a constant heating rate of 10 °C/min. TEG characterization was conducted under various temperature gradients. The cold side was kept at room temperature, and the hot side was controlled using a Peltier module connected to a DC power supplier (UP-3005T, PNCYS Co., Uiwang, Republic of Korea). The current, voltage, and power output of the TEGs were measured using a Keithley 2401 source meter (Tektronix, Beaverton, OR, USA). The practical temperature of the TEGs was recorded using K-type thermocouples.

## 3. Results and Discussion

**Figure 1:**
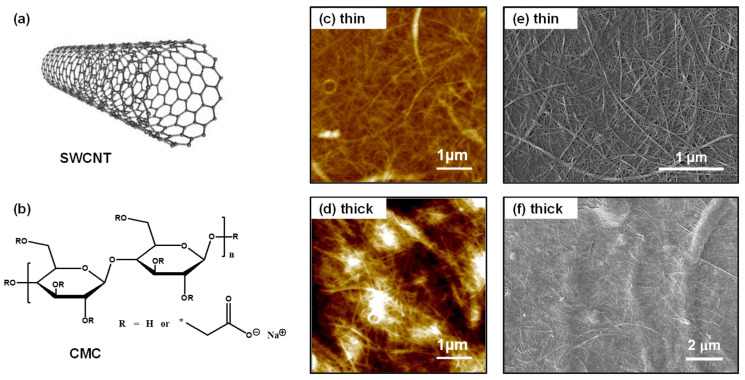
Chemical structure of (**a**) SWCNT and (**b**) CMC. Atomic force microscopy images of (**c**) thin and (**d**) thick CMC/SWCNT films (with a thickness of ≈100 nm and 5 µm, respectively), deposited from the composite dispersions in water. Scanning electron microscopy images of (**e**) thin and (**f**) thick CMC/SWCNT films, deposited from the composite dispersions in water.

Figure 1a,b display the chemical structures of SWCNT and CMC, respectively. SWCNTs generally give better *σ* compared with double- and multi-walled CNTs, based on the distinct one-dimensional structure of SWCNTs with high length-to-diameter ratio, which is advantageous for efficient charge carrier transport. This can potentially lead to high *PF* and also, large short-circuit current with reduced Joule heating in TEGs. This CMC polymer with sodium carboxylate functional groups is a water-soluble polyelectrolyte that can effectively wrap around SWCNTs, resulting in stable dispersion in aqueous solutions [35,36]. The hydroxyl and carboxylate groups in the CMC can chemically interact with SWCNTs through van der Waals forces and hydrogen bonding, which stabilizes the SWCNT dispersions and prevents strong π–π aggregation [27,36].

Using the dispersion-stable CMC/SWCNT composite solution, we prepared thin and thick films via spin-coating and drop-casting methods, respectively. The thickness of the thin and thick films was approximately 100 nm and 5 µm, respectively. First, we investigated the morphological characteristics of the thin/thick CMC/SWCNT films prepared from the composite solutions. As shown in the AFM topography images of the thin and thick films (Figure 1c,d), both samples exhibited smooth surface features with a root-mean-square roughness of ≈12 nm and 28 nm, respectively. This variation in roughness is likely to have occurred because the spin-casting method generally produces much more uniform film quality than drop-casting based on slow solvent evaporation. The cross-sectional SEM images shown in Figure S1 further confirm the uniform microstructure of the CMC/SWCNT thick films over a wide area. We found that the typical fibrillar network structures of the SWCNTs are well developed densely from the composite solutions, which could ensure promising electrical properties of CMC/SWCNT films. It is well known that the electrical performance of CNT derivatives is closely linked with their resulting nano/micro morphologies [17]. The SEM images in Figure 1e,f represent the nanofibrillar networks of the composite films with excellent film uniformity, in good agreement with the observations in the AFM analysis. In addition, we can see that the CMC matrix polymers fill and cover the voids between the SWCNT bundles with a high aspect ratio, which could be advantageous for effective phonon scattering and thus, low *κ*.

**Figure 2:**
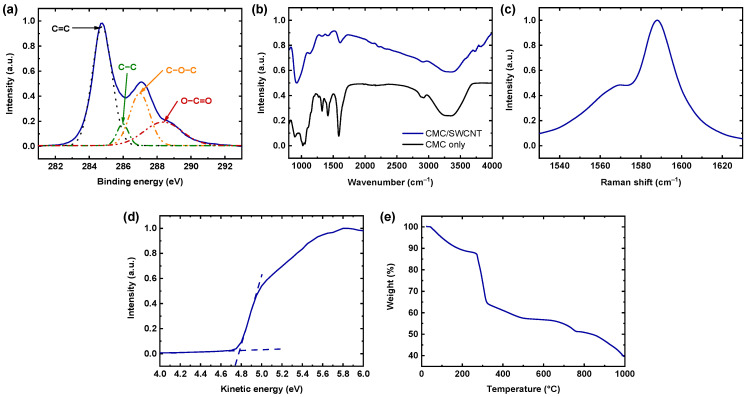
(**a**) X-ray photoelectron spectroscopy (C 1s regions), (**b**) Fourier transform infrared spectroscopy, (**c**) Raman spectroscopy, (**d**) ultraviolet photoelectron spectroscopy (secondary electron cutoff region) spectra, and (**e**) thermogravimetric analysis curve for CMC/SWCNT composite thin films.

The elemental composition of the CMC/SWCNT films was examined through various spectroscopic analyses. Figure 2a shows the high-resolution C 1s X-ray photoelectron spectroscopy spectrum for the CMC/SWCNT composite. The raw spectrum has been deconvoluted into four contributions by Lorentzian fitting. The peaks based on carbon–oxygen bonds (i.e., C−O−C and O−C=O) are indicative of the introduction of CMC polyelectrolytes in the composite. The carbon–carbon double bond peak (C=C) originates from the π-conjugated structure of SWCNTs. The Fourier transform infrared spectroscopy analysis (Figure 2b), sensitive to vibrational transitions of the polar bonds, also proved the presence of CMC component. The peaks occurring at 1587, 2905, and 3338 cm^−1^ for the CMC-only film, corresponding to the stretching vibrations of C=O, C–H, and O–H in CMC, respectively, accorded with the peaks at those wavenumbers for CMC/SWCNT composite film. Raman spectroscopy analysis for sensitively identifying non-polar bonds was implemented to further investigate the C=C bonds in the CMC/SWCNT (Figure 2c). The G-band of the SWCNT, resulting from in-plane vibrations of sp²-hybridized carbon atoms, appeared at 1587 cm^−1^ and 1569 cm^−1^ (corresponding to the G+ and G− peaks, respectively), indicating the present of SWCNTs in the composite films. In addition, we conducted ultraviolet photoelectron spectroscopy measurement to determine the electronic structure of the CMC/SWCNT composite films. Figure 2d presents the secondary electron cutoff region of the spectrum. We determined the kinetic energy of the CMC/SWCNT film based on the difference between the HeI photon energy (21.22 eV) and the threshold binding energy [37]. According to the linear extrapolation to zero kinetic energy for the abrupt drop observed in low kinetic energy, the work function of CMC/SWCNT films was determined as 5.0 eV, indicating the p-type feature of the CMC/SWCNT composite.

The thermal stability of the CMC/SWCNT composite films was evaluated through TGA, and the resulting weight-loss curve is shown in Figure 2e. The TGA curve demonstrated multistep weight-loss behavior, indicating the distinct decomposition processes of the CMC/SWCNT. The initial weight loss, occurring below approximately 150 °C, can be attributed to the evaporation of residual moisture and adsorbed water in the composite. A significant weight reduction was observed between 200 °C and 400 °C, corresponding to the decomposition of the CMC matrix, consistent with the thermal degradation of organic polymers. Above 600 °C, the rate of weight loss became slower, which may have been due to the gradual oxidation of carbon materials, including SWCNTs. At 1000 °C, the composite retained approximately ≈40% of its original weight, indicating the thermal stability of the SWCNTs. These findings confirm that CMC/SWCNT composite films exhibited good thermal stability, making them suitable for potential TE applications under moderate temperature conditions.

**Figure 3:**
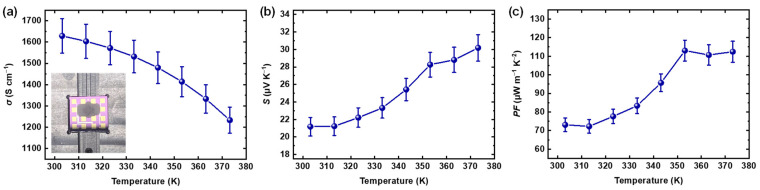
(**a**) *σ*, (**b**) *S*, and (**c**) *PF* plots for CMC/SWCNT composite films, as a function of temperature. The inset picture in (**a**) displays the sample prepared on a prepatterned chip for in-plane measurements.

Next, we evaluated the TE properties of the CMC/SWCNT composite films. Figure 3a–c show the *σ*, *S*, and *PF* plots as a function of temperature ranging from 303.15 K to 373.15 K. The *σ* and *S* of the samples were both measured through in-plane direction under conditions of high vacuum. The sheet resistance and resistivity of the sample used for TE property measurements were 4.06 ohm sq^−1^ and 6.13 × 10^−4^ ohm cm, respectively. The thickness of the samples was approximately 1.5 µm. The CMC/SWCNT film exhibited an average *σ* of 1630 S cm^−1^ at 303.15 K, decreasing to 1230 S cm^−1^ as the temperature increased to 373.15 K. This can be attributed to the metallic nature of the heavily doped SWCNTs. The *S* of the CMC/SWCNT was 21 µV K^−1^ at 303.15 K and gradually increased and reached 30 µV K^−1^ at 373.15 K; thus, the positive *S* values in this temperature range prove the p-type characteristics of the CMC/SWCNT composite. This trend reflects the well-known inverse relationship between *σ* and *S*, where an increase in carrier concentration enhances *σ* but suppresses *S*, and vice versa [38]. Such a trade-off is a fundamental limitation of TE materials, as it directly impacts optimization of the *PF*. Despite this inherent trade-off, the CMC/SWCNT composite films in the current study provided an average *PF* of 73 µW m^−1^ K^−2^ at near-room temperature, becoming almost saturated above 353 K with a maximum *PF* of 113 µW m^−1^ K^−2^. These results highlight the delicate balance required to maximize the *PF* while managing the interplay between *σ* and *S*. While the *PF* of our composite may not surpass that of some state-of-the-art TE materials, it is noteworthy that CMC/SWCNT composites have demonstrated competitive performance comparable to other solution-processed CNT-based composites [39,40,41]. The performance of our composite is therefore on par with these systems while maintaining excellent solution processability, making it suitable for scalable fabrication and deformable TE applications.

**Figure 4:**
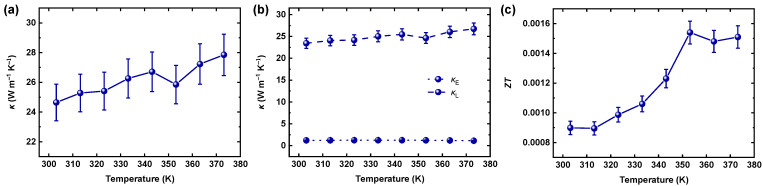
(**a**) *κ*, (**b**) *κ*_E_/*κ*_L_, and (**c**) *ZT* plots for CMC/SWCNT composite films, as a function of temperature.

The *κ* characteristics of the CMC/SWCNT films were studied, as shown in Figure 4a,b. The *κ* analysis was also conducted through the in-plane direction, similar to the *σ* and *S* measurements, based on a chip-based 3ω method [42]. It is well known that pure SWCNT derivatives intrinsically possess ultrahigh *κ* because of low phonon scattering, based on their quasi-one-dimensional structures [43,44,45]. The CMC/SWCNT films exhibited *κ* values around 25 to 28 W m^−1^ K^−1^ in this temperature range, much lower than the *κ* of SWCNTs in general. This observation indicates that the introduction of CMC effectively reduced the *κ* of the SWCNTs. The *κ* of the CMC/SWCNT composites was significantly lower than that of pure SWCNTs. This reduction can be attributed to the introduction of the insulating CMC matrix, which disrupts efficient phonon transport. The interfaces between SWCNTs and the CMC matrix create additional phonon scattering sites, impeding thermal conduction. Furthermore, the intrinsically low *κ* of the CMC further contributes to the reduced overall *κ* of the composite. These combined factors resulted in the observed decrease in *κ*. We further examined the electronic and lattice contributions (i.e., *κ*_E_ and *κ*_L_, respectively) in the composite system (Figure 4b). The *κ*_E_ was determined based on the Wiedemann–Franz law, *κ*_E_ = *LσT*, where *L* is the Lorentz factor. In this study, we assumed that the *L* of the CMC/SWCNT composite was equal to *L*_0_ = 2.44 × 10^−8^ V^2^ K^−2^, corresponding to a free electron gas, as commonly applied in similar systems [46,47]. As reported previously in the literature, the total *κ* of SWCNTs is mainly governed by *κ*_L_, due to the efficient phonon transport facilitated by strong carbon–carbon bonds, low defect density, and high phonon velocity [46]. While this assumption is widely accepted, we acknowledge that deviations in the *L* could influence the accurate evaluation of *κ*_E_, and further investigations may provide deeper insights into this aspect. In this context, the *κ*_L_ (23.4 W m^−1^ K^−1^ at 303.15 K) of the CMC/SWCNT composite was much greater than its *κ*_E_ (1.2 W m^−1^ K^−1^ at 303.15 K). Figure 4c plots the resulting *ZT* calculated using the *σ*, *S*, and *κ* at each temperature; consequently, a maximum *ZT* of 1.54 × 10^−3^ at 353.15 K was achieved from the water-processed CMC/SWCNT films.

**Figure 5:**
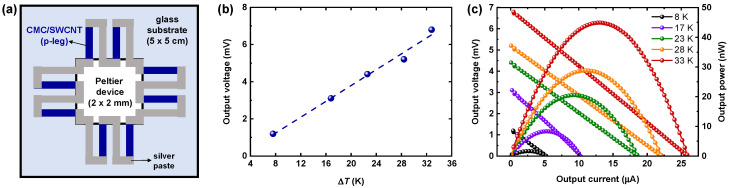
Demonstration of CMC/SWCNT-based TEGs. (**a**) Schematic device configuration for the TEG with 8 legs of water-processed CMC/SWCNT film. (**b**) Output voltage versus Δ*T* curve of CMC/SWCNT-based TEG. (**c**) Output voltage and output power as a function of output current under various steady-state Δ*T*.

Finally, to demonstrate this material’s practical energy-harvesting application, we fabricated organic TEGs using CMC/SWCNT films as water-processed p-type legs (without n-type counterparts). Figure 5a shows the device configuration of our TEG, in which eight legs of p-type CMC/SWCNT film are all connected electrically in series with silver paste. To build up the temperature gradient (Δ*T*), a Peltier device was placed at the center of the TEG and the edge was kept at room temperature. We first monitored the output thermal voltage changes as a function of Δ*T*, as plotted in Figure 5b. We observed that the resulting thermovoltage was linearly proportional to the applied Δ*T* across the whole range, suggesting the reliable operation of our TEGs. We next measured output voltage and power as a function of output current under various steady-state Δ*T* (Figure 5c). The open-circuit voltage (*V*_oc_) was determined through the relationship *V*_oc_ = *NS*Δ*T*, where *N* is the number of p-legs [48]. The TEG provided an average *V*_oc_/Δ*T* value of ≈185 μV K^−1^ in this Δ*T* range, nearly eight times the *S* of the CMC/SWCNT films. Consequently, we were able to obtain a maximum output power (*P*_max_) of ≈45 nW under a Δ*T* of 33 K, with a short-circuit current (*I*_sc_) and *V*_oc_ of 26 μA and 6.8 mV, respectively, corresponding to the expected *P*_max_ based on the relationship *P*_max_= *I*_sc_*V*_oc_/4. In addition, the introduction of an n-type counterpart is expected to significantly improve the TEG’s performance by forming a complementary p–n junction, thereby enhancing the overall efficiency and increasing the feasibility of scaling up the device for practical applications.

## 4. Conclusions

In summary, we implemented TE studies on water-processable CMC/SWCNT composite. The AFM and SEM microscope analyses proved that the CMC/SWCNT composite produced uniform and smooth films through dispersion in water, based on the incorporation of CMC matrix polyelectrolyte, ensuring the reliable TE performance of the composite films. The composite films exhibited a high *σ* of ≈1600 S cm^−1^ with an *S* of 21 µV K^−1^, resulting in a low temperature *PF* of 73 μW m^−1^ K^−2^ (a maximum *PF* of 113 μW m^−1^ K^−2^ at 353 K). In comparison with the *κ* of pure SWCNTs, the CMC-incorporated SWCNT films had a much lower *κ* of ≈25 W m^−1^ K^−1^ (at 303.15 K), beneficial for improving the *ZT* characteristics of the SWCNTs and thus, leading to a maximum *ZT* of 1.54 × 10^−3^ at 353.15 K. Furthermore, we successfully demonstrated water-processed organic TEGs using the composite films as a p-type component, which provided a *P*_max_ of ≈45 nW at a Δ*T* of 33 K. 

The integration of CMC/SWCNT composites with various printing techniques (e.g., inkjet printing, screen printing, and roll-to-roll processing) represents a promising direction for future research. Exploring these methods could enable the scalable and cost-effective fabrication of printed TEGs with tailored designs for specific applications, such as wearable electronics and IoT sensors.

## Data Availability

The original contributions presented in the study are included in the article. Further inquiries can be directed to the corresponding authors.

## Supplementary Materials

The following supporting information can be downloaded at https://www.mdpi.com/article/10.3390/ma18020337/s1. Figure S1. Cross-sectional SEM images for μm-thick CMC/SWCNT film prepared via ion milling.
