# Supplementary material for: Dispersion-Stable Carboxymethyl Cellulose/Single-Walled Carbon Nanotube Composite for Water-Processed Organic Thermoelectrics
Source: Materials (Basel). 2025 Jan 13;18(2):337. doi: 10.3390/ma18020337 (PMC11766926; doi:10.3390/ma18020337)
Supplement: Supplementary file 1 [file materials-18-00337-s001.zip › materials-3395373-supplementary.pdf]

Supplementary Information

Dispersion-Stable Carboxymethyl Cellulose/Single-Walled Carbon Nanotube Composite for Water-Processed Organic Thermoelectrics

Jaehee Jang,[‡a] Hyejeong Yeom,[‡a] Sujong Chae,[*b] Seyoung Kee[*a]

[a]Department of Polymer Engineering, Pukyong National University, Busan 48513, Republic of Korea.
[b]Division of Applied Chemical Engineering, Pukyong National University, Busan 48513, Republic of Korea

[‡]These authors contributed equally to this work.

E-mail: schae@pknu.ac.kr (S.C.); sykee@pknu.ac.kr (S.K.)

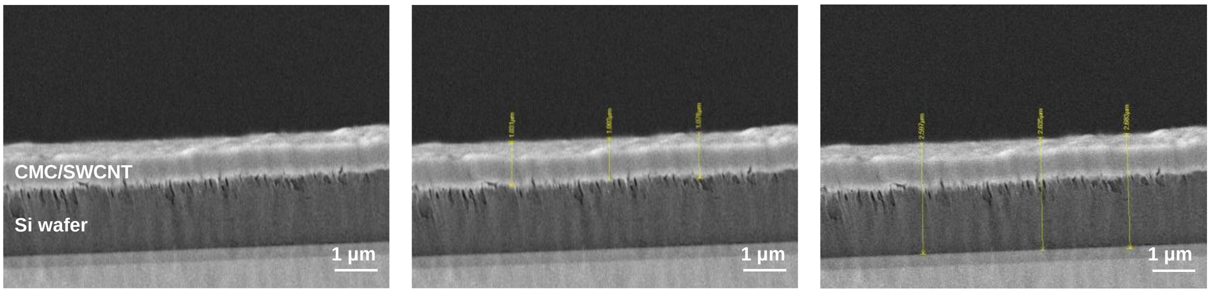

Figure S1. Cross-sectional SEM images for μm-thick CMC/SWCNT film prepared via ion milling.
